# Early transmissibility assessment of the N501Y mutant strains of SARS-CoV-2 in the United Kingdom, October to November 2020

**DOI:** 10.2807/1560-7917.ES.2020.26.1.2002106

**Published:** 2021-01-07

**Authors:** Kathy Leung, Marcus HH Shum, Gabriel M Leung, Tommy TY Lam, Joseph T Wu

**Affiliations:** 1 WHO Collaborating Centre for Infectious Disease Epidemiology and Control, School of Public Health, LKS Faculty of Medicine, The University of Hong Kong, Hong Kong SAR, China; 2 Laboratory of Data Discovery for Health (D^2^4H), Hong Kong Science Park, New Territories, Hong Kong SAR, China; 3 State Key Laboratory of Emerging Infectious Diseases, School of Public Health, The University of Hong Kong, Hong Kong SAR, China; 4 Joint Institute of Virology (Shantou University and The University of Hong Kong), Guangdong-Hongkong Joint Laboratory of Emerging Infectious Diseases, Shantou University, Shantou, China

**Keywords:** SARS-CoV-2, COVID-19, N501Y, lineage B.1.1.7, 20B/501Y.V1, VOC-202012/01, spike protein, fitness, transmissibility, United Kingdom

## Abstract

Two new SARS-CoV-2 lineages with the N501Y mutation in the receptor-binding domain of the spike protein spread rapidly in the United Kingdom. We estimated that the earlier 501Y lineage without amino acid deletion Δ69/Δ70, circulating mainly between early September and mid-November, was 10% (6–13%) more transmissible than the 501N lineage, and the 501Y lineage with amino acid deletion Δ69/Δ70, circulating since late September, was 75% (70–80%) more transmissible than the 501N lineage.

Two new severe acute respiratory syndrome coronavirus 2 (SARS-CoV-2) lineages carrying the amino acid substitution N501Y in the receptor-binding domain (RBD) of the spike protein have spread rapidly in the United Kingdom (UK) during late autumn 2020. Assessing the public health threat of these lineages (e.g. the potential for them to increase herd immunity thresholds if they displace other circulating SARS-CoV-2 strains) requires quantification of their comparative transmissibility. Here we adopted our previous epidemiological framework for relative fitness inference of co-circulating pathogen strains, which has been applied on influenza viruses [1] and SARS-CoV-2 D614G strains [2], to characterise the comparative transmissibility of the 501Y lineages.

## Severe acute respiratory syndrome coronavirus 2 501Y Variant 1 and Variant 2

The earlier 501Y lineage (501Y Variant 1) co-circulated with the 501N lineage between early September and mid-November in Wales, where its proportion never exceeded 2% among sequenced samples. However, a later 501Y lineage (501Y Variant 2, also named as B.1.1.7 by COVID-19 Genomics Consortium UK (CoG-UK) [3], 20B/501Y.V1 by Nextstrain (https://nextstrain.org/) and VOC-202012/01 by Public Health England [4]) started co-circulating with the 501N lineage in England in late September and became the dominant lineage in December. In the UK, the proportion of the 501Y Variant 2 lineage has increased from 0.1% in early October to 49.7% in late November among sequences available on GISAID (www.gisaid.org) as at 19 December 2020.

The proportion of 501Y Variant 2 has been growing rapidly, particularly in the South East, East of England and London regions since November [4,5], which suggests it may have a transmission advantage over the 501N lineage. Of note, 501Y Variant 2 is defined by an unusually large number of genetic changes, with at least 24 mutations including 14 non-synonymous mutations, four deletions and six synonymous mutations in ORF1ab, ORF8, nucleocapsid and spike proteins (Table).

**Table:** Genetic changes that characterise 501Y Variant 1 and Variant 2^a^ and occurred in the genetic branches preceding their lineages

Gene	501Y Variant 1	501Y Variant 2^a^
Spike	**N501Y**	H69, V70 deletion
		Y144 deletion
		**N501Y**
		A570D
		P681H
		T716I
		S982A
		D1118H
ORF1ab	S944L	T1001I
	H2357Y	A1708D
	P3395L	I2230T
	M6723I	S3675, G3676, F3677 deletion
ORF7a	T14I	–
ORF8	–	Q27 stop
		R52I
		Y73C
Nucleocapsid	–	D3L
		S235F

Only amino acid changes are shown.

^a^ 501Y Variant 2 was also named B.1.1.7 by COVID-19 Genomics Consortium UK (CoG-UK) [3], 20B/501Y.V1 by Nextstrain (https://nextstrain.org/) and VOC-202012/01 by Public Health England [4].

The most concerning mutation is indicated in bold.

The most concerning mutation is N501Y, which co-occurs with several mutations of potential biological importance, including P681H and deletion of the amino acid at the 69th and 70th residues (Δ69/Δ70) on the spike protein (Supplementary Table S1). Structural biological studies of the SARS-CoV-2 RBD offer insights proposing that 501Y may increase human angiotensin-converting enzyme 2 (ACE2) binding [6,7] and that the open conformation of the 501Y spike protein [8] is associated with more efficient viral entry and infection. Epidemiologically, however, there has been limited assessment to date investigating whether any of these mutations may have affected transmissibility [9].

## Reconstructing the phylogeny of 501N, 501Y Variant 1 and 501Y Variant 2

We downloaded the multiple sequence alignment of complete (or nearly complete) genomes of SARS-CoV-2 from the GISAID database initially on 14 December. To include more sequences for the study, we extended our search for 501N and 501Y sequences in the GISAID dataset downloaded on 19 December, including both the complete genomes and partial ones covering spike genes.

**Figure 1:**
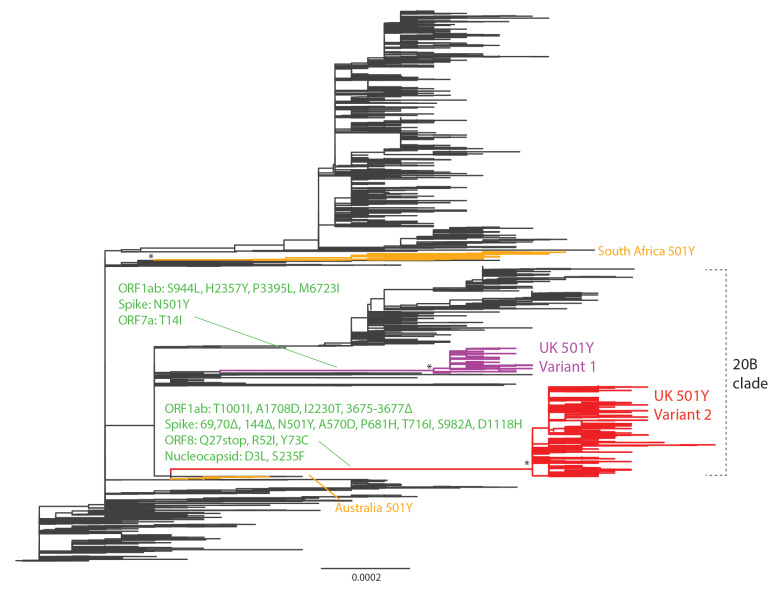
Phylogeny of SARS-CoV-2 showing the emergence of 501Y lineages, United Kingdom and other regions, as at 19 December 2020

We extracted all viral genomes carrying 501Y in the translated spike protein and analysed them with other closely related virus strains (identified through basic local alignment search tool (BLAST) search) in the global phylogeny (Supplementary Table S2). The resultant phylogeny built with the maximum likelihood method and generalised time-reversible (GTR) substitution model using FastTree version 2.1 [10] is shown in Figure 1. It indicates that the recent 501Y strains in the UK, since August/September 2020, emerged from the 20B clade (Nextstrain nomenclature) and formed two lineages. Both lineages have clear geographical separation in Wales vs England. The first 501Y lineage (501Y Variant 1) appeared in Wales in early September and persisted through November. The second 501Y lineage (501Y Variant 2, also named B.1.1.7, 20B/501Y.V1 and VOC-202012/01) appeared in England in late September and largely expanded to become the predominant lineage in the region since late November. Globally, two other lineages with 501Y (without Δ69/Δ70) have been detected in Australia and South Africa, circulating from June to July and October to November 2020, respectively.

Nt: nucleotide; SARS-CoV-2: severe acute respiratory syndrome coronavirus 2; UK: United Kingdom; US: United States.

The maximum likelihood tree was built from 7,003 genome nt sequences of SARS-CoV-2. The UK 501Y Variant 1 and 2 lineages are coloured in purple and red, respectively. The South African and Australian 501Y lineages are in orange. Amino acid changes at the preceding branches of UK 501Y Variant 1 and 2 are indicated in green. Some 501Y variants in sporadic emergence (including those in Spain and the US, etc.) without establishment of a lineage of more than 10 sequences are not shown. The asterisks refer to the > 0.98 topology supports (Shimodaira-Hasegawa test) for the monophyletic grouping of the 501Y lineages. Branch scale is shown at the bottom of the tree in the unit of substitutions per site.

## Comparative transmissibility of 501Y Variant 1 and Variant 2

We assumed that the N501Y mutation and Δ69/Δ70 deletions characterise the three strains (501N, 501Y Variant 1 and 501Y Variant 2), but their differential transmissibility (if any) might be attributable to the combination of N501Y and other mutations acquired in the emergence of 501Y Variant 1 and 2 lineages (Table and Supplementary Table S1). For conciseness, we used *N*, *Y*1 and *Y*2 to denote the three strains. We defined the comparative transmissibility of any two strains as the ratio of their basic reproductive numbers$\;(R_{0})$. That is, the comparative transmissibility of strains *Y*1 and *Y*2 with respect to strain *N* was $\sigma_{Y1}=R_{0}^{Y1}/R_{0}^{N}$ and $\sigma_{Y2}=R_{0}^{Y2}/R_{0}^{N}$, respectively.

We extended the previous competition transmission model of two viruses [1,2] and applied the fitness inference framework to the sequence data collected from the UK between 22 September and 16 November 2020, during the co-circulation period of the three strains (see Supplementary Material for details). The inference framework incorporates both incidence and genotype frequency data that reflect the local comparative transmissibility of co-circulating strains. Using confirmed deaths (adjusted for the delay between symptom onset and death [11]) as the proxy for the coronavirus disease (COVID-19) epidemic curve [12], we estimated that $\sigma_{Y1}$ was 1.10 (95% credible interval (CrI): 1.06–1.13) and $\sigma_{Y2}$ was 1.75 (95% CrI: 1.70–1.80). That is, the $R_{0}$ of the 501Y Variant 1 and Variant 2 was 10% (95% CrI: 6–13%) and 75% (95% CrI: 70–80%) higher, respectively, than that of the 501N strain.

**Figure 2:**
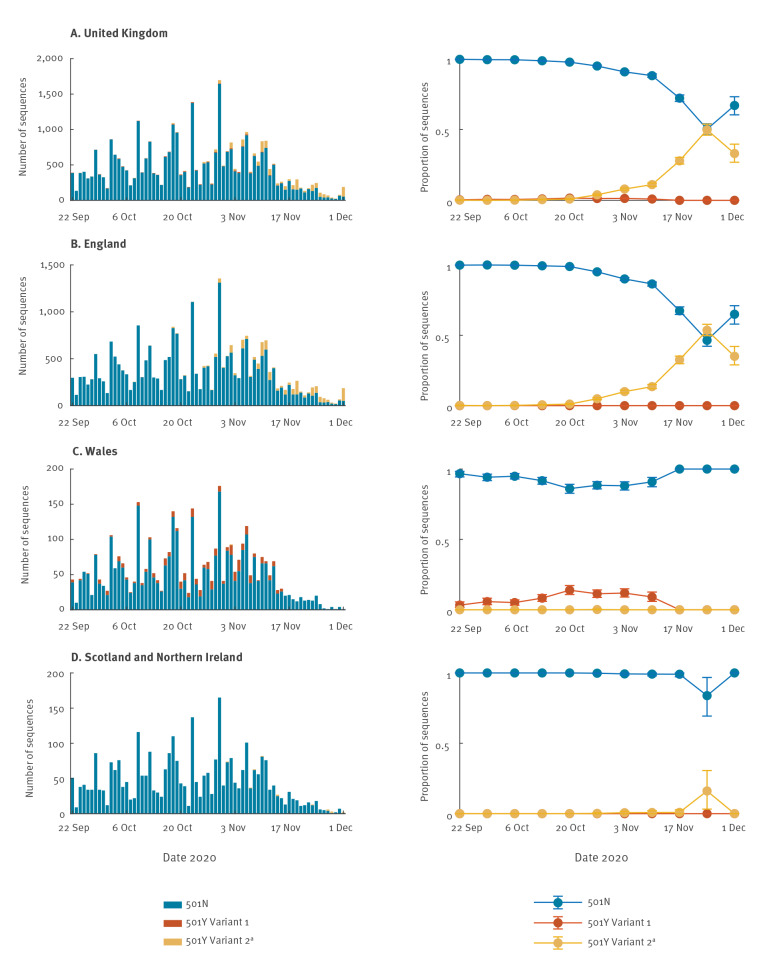
Observed daily number and weekly proportion of SARS-CoV-2 501N, 501Y Variant 1 and 501Y Variant 2^a^ sequences during their co-circulation, by dates of sampling, (A) United Kingdom, (B) England, (C) Wales, (D) Scotland and Northern Ireland, 22 September–1 December 2020

CI: confidence interval; SARS-CoV-2: severe acute respiratory syndrome coronavirus 2; UK: United Kingdom.

^a^ 501Y Variant 2 was also named B.1.1.7 by COVID-19 Genomics Consortium UK (CoG-UK) [3], 20B/501Y.V1 by Nextstrain (https://nextstrain.org/) and VOC-202012/01 by Public Health England [4].

**Figure 3:**
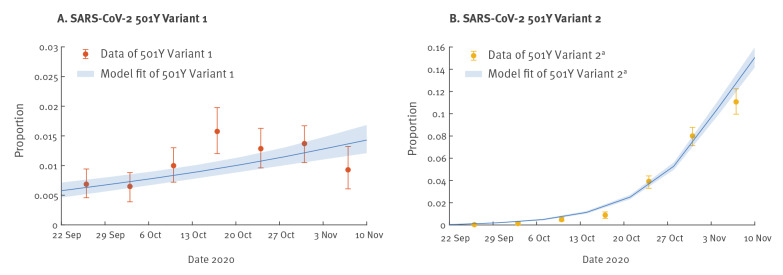
Observed and fitted proportion of SARS-CoV-2 (A) 501Y Variant 1 and (B) 501Y Variant 2^a^ sequences during their co-circulation with SARS-CoV-2 501N, United Kingdom, 22 September–16 November 2020

CI: confidence interval; COVID-19: coronavirus disease; CrL: Credible interval; SARS-CoV-2: severe acute respiratory syndrome coronavirus 2; UK: United Kingdom.

^a^ 501Y Variant 2 was also named B.1.1.7 by COVID-19 Genomics Consortium UK (CoG-UK) [3], 20B/501Y.V1 by Nextstrain (https://nextstrain.org/) and VOC-202012/01 by Public Health England [4].

The fitted model was largely congruent with the observed proportions of the three strains over time, except during 13–19 October and 3–9 November, for 501Y Variant 1 (Figure 2-3). Since 501Y Variant 1 mainly co-circulated with 501N in Wales, we also performed a separate analysis using sequence data from Wales only. We estimated $\sigma_{Y1}$ was 1.14 (95% CrI: 1.11–1.19) but were not able to estimate $\sigma_{Y2}$ because there were only two 501Y Variant 2 sequences sampled before 30 November from Wales in our dataset.

The circles and error bars indicate the observed proportion, with 95% multinomial CIs among sequence data.

The time series of confirmed COVID-19 deaths in the UK was used in the estimation of comparative transmissibility. The circles and error bars indicate the observed proportion, with 95% multinomial CIs among sequence data. The blue lines and shades indicate the posterior mean and 95% CrI of the estimated proportions of 501Y lineages.

### Sensitivity analyses to assess the possible impact of generation times on findings

We conducted a sensitivity analysis to assess the possibility that the transmission advantages of 501Y lineages were due to shorter generation time [2]. Assuming the same $R_{0}$ for the three strains, we estimated the mean generation time of 501Y Variant 2 was 44% (95% CrI: 39–47%) shorter than that of 501N, but the inference failed to converge to generate estimates for 501Y Variant 1. Moreover, this fitted model had significantly higher Akaike information criterion (AIC) than our base case model, hence favouring our base case conclusion that the transmission advantage of 501Y Variant 2 was due to higher $R_{0}$ but not shorter generation time.

## Discussion

Our findings indicate that 501Y Variant 2 (also named B.1.1.7, 20B/501Y.V1 and VOC-202012/01) is estimated to present an $R_{0}$ 1.75 times higher than 501N, meaning it is 75% more transmissible compared with the 501N strain. Of note, this variant has also become the dominant strain in England in November/December 2020. These observations would imply more rapid and stringent control measures would be necessary to suppress spread, which is precisely what the UK government effected on 19 December, including the addition of a new tier 4 set of restrictions [13]. In addition, a number of countries closed their borders to travellers from the UK.

As at 19 December 2020, 501Y Variant 2 cases had been identified outside of the UK in 21 countries and regions including Denmark, Hong Kong, Italy, Japan, Spain, Singapore and the United States (US) [14]. It remains unclear whether they correspond to exportation from the UK or local spread until more historical sequence data become available. Although sporadic spread of SARS-CoV-2 variants with the 501Y mutation occurred in Wales and elsewhere (e.g. Australia, Spain and the US), not all variants with 501Y have become prominent. On the other hand, in South Africa, a new variant with 501Y but not Δ69/Δ70 has emerged and spread rapidly since late October [15]. Our phylogenetic analyses show that the South African variant is genetically distant and has many mutations not shared with 501Y Variant 2. With only limited sequence data, we were not yet able to accurately quantify the comparative transmissibility of the South African variant. However, if this variant were also more transmissible, more studies would be necessary to investigate the multiple non-synonymous mutations shared or not shared with 501Y Variant 2, as well as how these mutations (such as Δ69/Δ70 and P681H of the spike protein) may account for the increased transmissibility. Future studies of their individual and combinatorial effects on the viral phenotypes are warranted.

Our study has several limitations. First, our comparative fitness analysis was based on the sequence data released in GISAID and is thus subject to the selection bias of sequences being released to the public database. The proportion of 501Y Variant 2 sequences after 16 November varied substantially by sampling time and location, even within England, and therefore we limited our analysis to the co-circulating period of the three strains between 22 September and 16 November 2020.

Second, we assumed that the three strains co-circulated locally during the study period, but our phylogenetic analyses suggest that 501Y Variant 1 and 2 have clear geographical separation in Wales vs England. Our estimation of comparative transmissibility should not be substantially affected if the $R_{0}$ of the comparator 501N variant remains the same. However, the effective reproductive number ($R_{eff}$) of 501N might be different in Wales and England because of different non-pharmaceutical interventions implemented in different locations (e.g. Tier 1–3 interventions) during the period studied. Therefore, it is urgent to compare our estimates of $\sigma_{Y1}\;$and $\sigma_{Y2}$ to observed serial intervals and $R_{eff}$ of 501Y Variant 2 from contact tracing results of cluster of cases.

Third, the currently available data did not allow us to explore whether age-specific susceptibility to infection was the same for the three strains. If the N501Y mutation would increase the binding to human ACE2, it might increase the susceptibility of children to 501Y Variant 2 [16].

Fourth, we assumed recovery from infection with any strain provided protection against re-infection of all strains during our study period, but 501Y Variant 2 carries an unusually large number of mutations and some of them, for example Δ69/Δ70 (Supplementary Table S1), might link to immunoescape, as was first identified in immunocompromised patients [17,18]. It is therefore unknown to what extent a person infected by one strain is protected against infection of another strain.

Furthermore, the model applied here did not consider viral importation. This is less problematic for 501Y Variant 1 and 2 because they form their own lineages with predominant samples from the UK, whereas 501N data are composed of multiple genetic lineages that might derive from importation; however, if so, the transmissibility of 501N would likely be overestimated, and the relative transmissibility of 501Y would be higher than the current estimate.

Further work should clarify the role, if any, of increased mobility and population mixing that may have been concurrent with the circulation of the 501Y Variant 2 in explaining higher transmissibility. In particular, this should be done through comparison with contact tracing results of clusters of cases of 501Y Variant 2 [19]. Assessment of clinical severity changes associated with the new variants would require several more weeks of close and careful observation [19,20]. Finally, given the numerous mutations associated with 501Y variants, and thus the potential for antigenic changes, intensified immunogenomic surveillance is necessary to identify instances of re-infection in previous confirmed COVID-19 patients, as well as breakthrough infections among those who have been vaccinated.

## Data sharing statement

We collated all data from publicly available data sources. All data included in the analyses are available in the main text or the supplementary materials.

## Supplementary Data

2983000 Supplementary Material
