# Addendum for Euro Surveill. 2021;26(1)

**DOI:** 10.2807/1560-7917.ES.2021.26.3.210121a

**Published:** 2021-01-21

**Authors:** Eurosurveillance editorial team

**Affiliations:** 1 European Centre for Disease Prevention and Control (ECDC), Stockholm, Sweden

In the article ‘Early transmissibility assessment of the N501Y mutant strains of SARS-CoV-2 in the United Kingdom, October to November 2020’ by K. Leung et al. published on 7 January 2021, the term ‘clade GR’ as per the GISAID Initiative on common clade nomenclature system was added to complete the different assignments of the newly detected SARS-CoV-2 variant at the time of publication. It was further specified that the sequences were retrieved from the GISAID Initiative’s EpiCoV database. The acknowledgement was replaced by ‘We gratefully acknowledge the following authors from the originating laboratories responsible for obtaining the specimens, as well as the submitting laboratories where the genome data were generated and shared via GISAID, on which this research is based. ’ to match the wording in the Supplementary material.

The table with sequence contributors in the Supplementary Table S2 was replaced by a table with more comprehensive information about submitters by authors.

These additions were made on 21 January 2021.

